# Exercise and time-restricted and/or dietary feeding jointly improve hepatic lipid homeostasis in diet-induced obese mice

**DOI:** 10.1038/s41598-026-45394-4

**Published:** 2026-03-25

**Authors:** Nicole Power Guerra, Anja U. Bräuer, Markus H. Gräler, Katharina Leyens, Brigitte Vollmar, Angela Kuhla

**Affiliations:** 1 Institute for Experimental Surgery, Rostock University Medical Center, Schillingallee 69a, 18057 Rostock, Germany; 2 Research Group Anatomy, School for Medicine and Health Science, Carl Von Ossietzky Universität Oldenburg, Oldenburg, Germany; 3 Research Center for Neurosensory Science, Carl Von Ossietzky Universität Oldenburg, Oldenburg, Germany; 4 Department of Anesthesiology and Intensive Care Medicine, Jena University Hospital, 07747 Jena, Germany; 5 Center for Molecular Biomedicine (CMB), Jena University Hospital, 07745 Jena, Germany; 6 Center for Sepsis Control and Care (CSCC), Jena University Hospital, 07747 Jena, Germany; 7 Centre for Transdisciplinary Neurosciences Rostock (CTNR), Rostock University Medical Centre, Gehlsheimerstraße 20, 18147 Rostock, Germany

**Keywords:** Diet-induced obesity, High-fat diet, Dietary change, Treadmill exercise, Time-restricted feeding, Nuclear receptors, Lipogenesis- and β-oxidation-related genes, Lipidomics, Diseases, Gastroenterology, Physiology

## Abstract

Obesity and metabolic syndrome are associated with dysregulated hepatic lipid metabolism, contributing to metabolic dysfunction-associated steatotic liver disease (MASLD). Though lifestyle interventions such as a low-fat diet (LFD), treadmill (TM) exercise, and time-restricted feeding (TRF) reduce hepatic lipid accumulation, their combined effects on hepatic lipid composition and lipid metabolism-related gene regulation remain poorly understood. Here, we examined the individual and combined effects of LFD, TM, and/or TRF on liver function, comprehensive hepatic lipidomics, and lipid metabolism-related gene expression in diet-induced obese mice, thereby extending our previous work through detailed lipid class-specific analyses and assessment of interactive intervention effects. Among all interventions, LFD led to the greatest weight loss and normalized plasma aspartate aminotransferase (AST) as well as alanine aminotransferase (ALT) levels. Combined interventions, including TM and TRF, reduced markers of liver damage even under continued HFD conditions compared to HFD alone. LFD with TRF and/or TM decreased the expression of lipogenic genes (*Srebf1*, *Lxrα*, *Apoe*), while expression of genes further involved in lipid synthesis (*Fasn* and *Hmgcr*) tended to be increased when TM was combined with either LFD or HFD. β-oxidation-related genes (*Ppara, Acox1, Cpt1a*) were most downregulated in the LFD groups vs. the HFD + TM group, likely representing a metabolic adaptation to increased lipid mobilization. For the first time, lipidomics analysis demonstrated that in particular LFD alone or in combination with TM most effectively increased sphingomyelin (SM) and dihydrosphingomyelin (DHSM) as well as lysophosphatidylcholine (LPC) and phosphatidylcholine (PC), potentially reflecting compensatory lipid remodeling. Taken together, these findings highlight distinct and additive effects of combined lifestyle interventions on hepatic lipid composition and gene regulation, clearly delineating the novel contributions of the present study and supporting combined dietary and physical strategies as potential approaches to improve hepatic lipid homeostasis and mitigate MASLD development.

**Supplementary Information:**

The online version contains supplementary material available at 10.1038/s41598-026-45394-4.

## Introduction

More than 40% of the global population is affected by overweight or obesity -a condition that has reached pandemic proportions^1^. As early as 1989, Kaplan described the “Deadly Quartet” -abdominal obesity, hypertension, hyperglycemia, and hypertriglyceridemia-now widely recognized as metabolic syndrome^2–4^. Obesity and metabolic syndrome are strongly associated with metabolic dysfunction-associated steatotic liver disease (MASLD), which is considered the hepatic manifestation of this syndrome^5–7^. The etiology of MASLD is complex and multifactorial. Key mechanisms contributing to hepatic lipid accumulation include increased influx of fatty acids from the diet or adipose tissue, enhanced de novo lipogenesis, reduced fatty acid β-oxidation, and impaired export of triglycerides (TGs) as very-low-density lipoproteins (VLDL)^8^. These processes are tightly regulated by nuclear receptors and their target genes.

One central regulator is liver X receptor alpha (LXRα), a transcription factor involved in de novo cholesterol synthesis^9^. Upon activation, LXRα induces the gene expression of fatty acid synthase (*Fasn*), leading to increased levels of the FAS enzyme, which catalyzes the conversion of acetyl-CoA and malonyl-CoA into fatty acids^10^. In parallel, sterol regulatory element-binding protein-1c (SREBP1c) upregulates expression of lipogenesis-related genes, including 3-hydroxy-3-methylglutaryl coenzyme A (*Hmg-CoA)* reductase (which corresponds to the murine terminology *Hmgcr*). The enzyme encoded by this gene, HMG-CoA reductase, is a key enzyme in cholesterol biosynthesis^11^. Cholesterol transport via high- and low-density lipoproteins is modulated by apolipoprotein E (ApoE) and related factors^12,13^. Counteracting these lipogenic pathways is peroxisome proliferator-activated receptor alpha (PPARα), a lipid-sensing nuclear receptor that promotes fatty acid oxidation. Activation of PPARα induces the expression of genes involved in mitochondrial and peroxisomal β-oxidation, such as acyl-coenzyme A oxidase 1 (*Acox1*) and carnitine palmitoyltransferases 1a and 2 (*Cpt1a, Cpt2a*)^14^, ultimately reducing hepatic and systemic TGs and cholesterol levels, indicative for enhanced lipolysis^15^.

Beyond classical lipids, emerging evidence highlights the relevance of bioactive lipid species -such as ceramides (Cer) and sphingolipids including sphingomyelins (SM), dihydrosphingomyelins (DHSM) and sphingosine- which are closely associated with obesity, insulin resistance, liver dysfunction, and altered lipid metabolism. These lipids are increasingly viewed as both biomarkers and potential contributors to the pathogenesis of metabolic syndrome and MASLD^16,17^. In addition, altered serum levels of bis(monoacylglycerol)phosphate (BMP) fatty acids have been observed in patients with metabolic dysfunction-associated (MASH) and liver cirrhosis^18^. Other lipid classes, such as lysophosphatidylcholines (LPC), lysophosphatidylethanolamines (LPE), and phospholipids like phosphatidylcholines (PC), are also implicated in oxidative stress and vascular dysfunction, partly through the release of unsaturated fatty acids, which may further aggravate metabolic disease^19,20^.

Lifestyle interventions -including dietary modification, physical activity, and intermittent fasting- remain the cornerstone of prevention and treatment for obesity-related metabolic disorders^21–26^. In line with these findings and based on our previous data indicating that a combined lifestyle approach can help prevent MASLD^27^, this study examines for the first time the potential combined effects of these interventions on hepatic lipid composition, including genes involved in both lipid synthesis and β-oxidation pathways.

## Methods

### Animals and experimental design

Ninety female C57BL/6 J mice, 4 weeks old, were purchased from Charles River (Sulzfeld, Germany) for the study. All animal experiments were approved by the local Animal Research Committee of the state of Mecklenburg-Western Pomerania (LALLF M-V/TSD/7221.3-2-001/18, approved on March 1, 2018), conducted in accordance with EU Directive 2010/63/EU and were complied with ARRIVE guidelines. The mice were housed in standard cages with five animals per cage under controlled environmental conditions (temperature: 21 ± 3 °C; 12/12-h light/dark cycle, with lights on from 6:00 AM to 6:00 PM). To induce obesity, all animals were fed a high-fat diet (HFD; D12492, Research Diets, New Brunswick, NJ, USA) ad libitum for the first six months. Following this induction phase, the cages were randomly assigned to six experimental groups (n = 15 per group), and different interventions were applied over the subsequent six months, as previously described by Power Guerra et al.^28^.

**Fig. 1:**
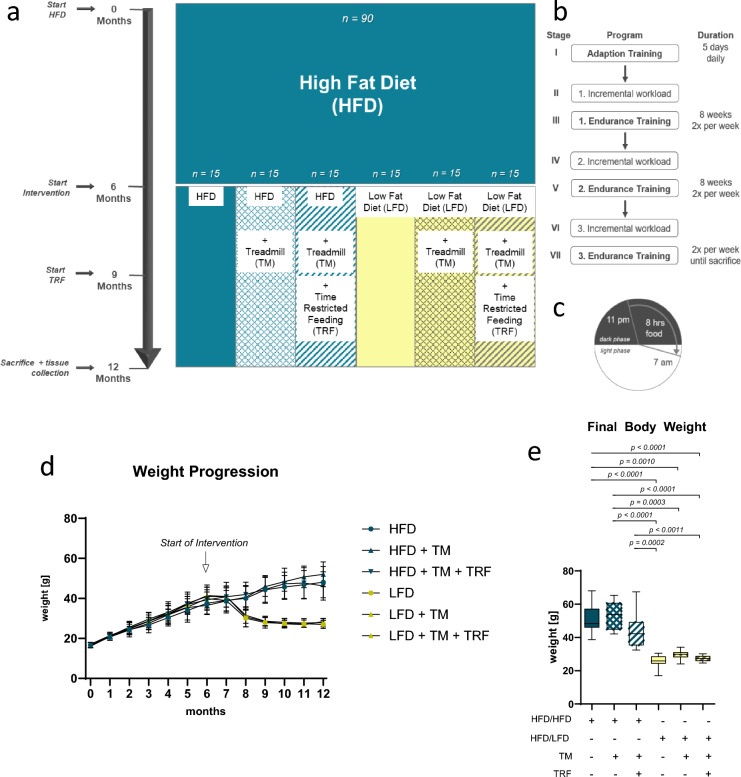
Illustration of the experimental setup (**a**) Adult female C57BL/6 J mice (n = 90) were fed a high-fat diet (HFD) for six months to induce obesity. They were then divided into six groups. Group 1 remained on HFD. Groups 2–6 received various interventions: treadmill exercise (TM) (HFD/HFD + TM), TM plus time-restricted feeding (TRF) (HFD/HFD + TM + TRF), a switch to low-fat diet (LFD; HFD/LFD), LFD plus TM (HFD/LFD + TM), and LFD with TM and TRF (HFD/LFD + TM + TRF), each with n = 15. After diet change, TM training began; TRF was introduced after three months. At the study’s end, mice were euthanized, and blood and liver samples were collected. The TM protocol (**b**) included seven phases with three endurance stages. An initial workload test determined each mouse’s maximum running speed. In TRF groups, food was available only during the nocturnal active phase (8 h feeding, 16 h fasting; (**c**) Body weights were tracked monthly (**d**) Of the initial 90 mice, 84 completed the study. Final weights were recorded before euthanasia (**e**, n = 84). Blue box plots indicate HFD groups, yellow indicate LFD groups. A table summarizes the interventions (“ + ” = applied, “−” = not applied). Statistical analysis used the Kruskal–Wallis test with Dunn’s post hoc test. Data are shown as mean ± SD; significance was set at p < 0.05. Figures 1a–c have been previously published^27,28^. Figures 1d and 1e use data from Power Guerra et al.^27,28^ for conceptual illustration of weight gain and final body weight and do not represent new measurements from the animals used in the present study.

The first group (HFD/HFD) continued on the HFD without additional intervention. The second group (HFD/HFD + TM) remained on the HFD and underwent treadmill (TM) exercise using a standardized protocol (TM 303401, TSE Systems Inc., Chesterfield, USA). The third group (HFD/HFD + TM + TRF) also performed TM, and time-restricted feeding (TRF) was introduced after three months. The fourth group (HFD/LFD) switched from the HFD to a low-fat diet (LFD; D12450J, Research Diets, New Brunswick, NJ, USA). The fifth group (HFD/LFD + TM) received the LFD and performed TM exercise. The sixth group (HFD/LFD + TM + TRF) received all three interventions: diet change to LFD, TM training, and TRF, with the latter introduced in the second half of the intervention period. Body weight was recorded weekly and immediately prior to sacrifice. An overview of the experimental design is presented in Fig. 1a.

The dietary intervention involved replacing the HFD (60% fat, 20% protein, 20% carbohydrates) with an LFD containing 10% fat, 20% protein, and 70% carbohydrates. Both diets were matched in lard and protein composition. TM was carried out in 60 mice, twice per week, following a previously established protocol^28^, as outlined in Fig. 1b. TM velocity was adjusted to match the speed of the slowest mouse, also to prevent overloading the animals or inducing excessive lactate accumulation. TRF was applied to 30 mice after the third month of TM exercise, following the same schedule described in earlier work^28^ and maintained for the remaining three months. Food was dispensed automatically using a modified autofeeder (EHEIM, Deizisau, Germany) with an enlarged opening, and food drops were monitored at 11:00 PM via an infrared webcam. At 7:00 AM, animals were transferred to fresh cages with water access but without enrichment (Fig. 1c). Figure 1a–c have been previously published^27,28^. Figure 1d and e present data from Power Guerra et al.^27,28^ for conceptual illustration of weight gain and final body weight. These panels do not represent new measurements from the animals used in the present study, but are included for conceptual illustration.

### Euthanasia of the mice and harvesting liver tissue

Under deep anesthesia with 5% isoflurane (Baxter, Unterschleißheim, Germany), 0.8 L/min O_2_ (Air Liquide, Hamburg, Germany) and 1.25 L/min N_2_O (Air Liquide, Hamburg, Germany) mice were humanely euthanized by exsanguination via retrobulbar blood collection, in accordance with institutional and national animal welfare guidelines. Blood was collected and processed as described by Power Guerra et al.^29^. A cervical dislocation was conducted postmortem to ensure death. Subsequently, a laparotomy was performed to harvest and weigh liver tissue. The liver was minced, flash-frozen in liquid nitrogen and stored at − 80 °C for RNA analysis and lipidomics.

### Assays

Liver damage was assessed by spectrophotometric measurement of plasma aspartate aminotransferase (AST), alanine aminotransferase (ALT) and albumin levels (Cobas c111; Roche Diagnostics, Mannheim, Germany) using commercial kits. Plasma levels of β-hydroxybutyrate were determined with assay kits according to the manufacturers’ instructions (Cayman Chemical, USA).

#### Quantitative real-time PCR

RNA isolation and reverse transcription into cDNA were performed as previously published^29^. mRNA expression analyses were performed via quantitative real-time PCR in BioRad iQ5 Multicolor Real Time PCR Detection System (Conquer Scientific, San Diego, CA, USA) with iQ™ SYBR® Green Supermix (Bio-Rad Laboratories, Munich, Germany). Primer sequences are shown in Table 1. Measurement results were normalized against the housekeeping gene 40S ribosomal protein S18 (*Rps18*) as well as positive control (PC, C57BL6 liver pool) and relative quantification was carried out via the 2^−ΔΔCT^ method (first Δ against *Rps18* Ct values and second Δ against PC Ct values).

**Table 1:** qRT-PCR primers for murine lipogenesis- and β-oxidation-related genes.

Transcript	forward primer (5′–3′)	reverse primer (5′–3′)
*Acox1*	GCTGTCTTCCTGCTGGGG	CCTGGTTTGGGGAGTCCTTC
*Ppara*	ACATTCGAGGCTCCAGTGAATTCGG	GGCAGGTCTACTTTGGAGTCATTGC
*Cpt1a*	CCCAAGCAATACCCAAAGAA	TTGTGAGGTGCTGATGTACCA
*Cpt2*	TCTGACCACAGTGAGGAATGTCCAC	TGGAGTCACAGAAGGAGTGGCTAAG
*Apoe*	GCCTTGCAGAAAAGAGAGCT	AAAGAAAGTCTTCACCTGGC
*Fasn*	TACCATGGCAACGTGACACT	TAGCCCTCCCGTACACTCAC
*Hmgcr*	CAG GAT GCA GCA CAG AAT GT	CTT TGC ATG CTC CTT GAA CA
*Sreb1f*	GTA CCT GCG GGA CAG CTT AG	CAG GTC ATG TTG GAA ACC AC
*Srebf2*	ACC TGT GAC CTG CTA CTG TC	CAG CTG GTG TGT ACG GGT AG
*Lxrα*	TGCCATCAGCATCTTCTCTG	GGCTCACCAGCTTCATTAGC
*Rps18*	AGGATGTGAAGGATGGGAAG	TTGGATACACCCACAGTTCG

### Lipidomics analysis from liver tissue

*Lipid extraction* Frozen liver tissue was transferred to glass centrifuge tubes containing chloroform, methanol, and 37% hydrochloric acid, supplemented with 1% butylated hydroxytoluene (BHT). The tissue was gently homogenized on ice. TopFluor lysophosphatidic acid (LPA, Avanti) was added as internal standard (1 µL per mg tissue), dissolved in chloroform. After adding chloroform and water, samples were vortexed and incubated for 30 min at room temperature in the dark. Samples were centrifuged (1260×*g*, 10 min, RT), and the organic phase was collected into new glass vials. Lipid extracts were stored overnight in a nitrogen chamber (6% O₂, dark) and kept at − 20 °C until analysis.

*Liquid chromatography coupled to triple quadrupole mass spectrometry (LC–MS/MS)* Lipid extracts from liver tissue were prepared as described above. After extraction and drying (SpeedVac), lipids were resuspended in methanol:chloroform (4:1). Internal standards (e.g., C17-LPC, C15-Cer, C17-SM, C17-S1P; Avanti) were used for quantification. Samples were analyzed using HPLC (Shimadzu) with a reverse-phase C18 column, and a gradient of methanol and formic acid in water. Lipids were detected using a QTrap triple quadrupole mass spectrometer (Sciex) in positive ion mode with ESI and APCI sources. Lipid quantification was performed using Analyst software based on internal and external standard curves. Lipid concentrations were expressed as pmol/mg of liver tissue. The results are visualized as heat maps illustrating relative lipid abundances, with white representing low and blue representing high values.

### Correlation analysis

Correlation analyses were performed using GraphPad Prism version 10.4.2. Pearson correlation was used to assess linear relationships between the within-group mean values of hepatic lipid classes (sphingolipids, lysophospholipids and phospholipids), liver injury markers, core lipogenic genes, and β-oxidation-related genes. All possible pairwise correlations among these parameters were calculated and visualized in a heat map. In the heat map, blue represents strong positive correlations (0.70–1.00), red represents strong negative correlations (-1.00 to -0.70), and lighter colors indicate moderate correlations (> 0.40 or < -0.40).

### Statistics

**Fig. 2:**
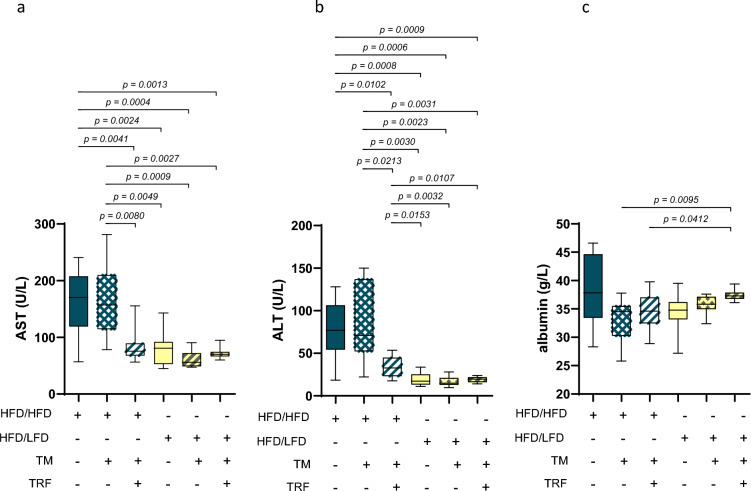
Plasma concentration of aspartate aminotransferase (AST), alanine aminotransferase (ALT), and albumin. AST (**a**: HFD/HFD n = 12, HFD/HFD + TM n = 12, HFD/HFD + TM + TRF n = 13, HFD/LFD n = 13, HFD/LFD + TM n = 15, HFD/LFD + TM + TRF n = 11), ALT (**b**: HFD/HFD n = 12, HFD/HFD + TM n = 12, HFD/HFD + TM + TRF n = 13, HFD/LFD n = 13, HFD/LFD + TM n = 15, HFD/LFD + TM + TRF n = 11) and albumin (**c**: HFD/HFD n = 12, HFD/HFD + TM n = 12, HFD/HFD + TM + TRF n = 13, HFD/LFD n = 13, HFD/LFD + TM n = 15, HFD/LFD + TM + TRF n = 11). Blue dots and box plots indicate HFD groups, yellow dots and box plots indicate diet change to LFD. The table below the figure displays the individual groups, respectively. Table is read from top to bottom, ‘ + ’ denotes implementation of a given diet or intervention and ‘−’ its absence. Significance was assessed using the Brown-Forsythe and Welch ANOVA followed by Tamhane’s multiple comparisons test (**a**: F value (F) = 20.35, degrees of freedom (DF) = 5, **b**: F = 22.80, DF = 5) and Kruskal–Wallis test followed by Dunn’s post hoc test (**c**). Data are presented as mean ± SD and statistical significance was set at p < 0.05. HFD = high-fat diet, LFD = low-fat diet, TM = treadmill, TRF = time-restricted feeding. The data for ALT, AST, and albumin have already been presented as a correlation matrix in Power Guerra et al.^27^ (own group data).

**Fig. 3:**
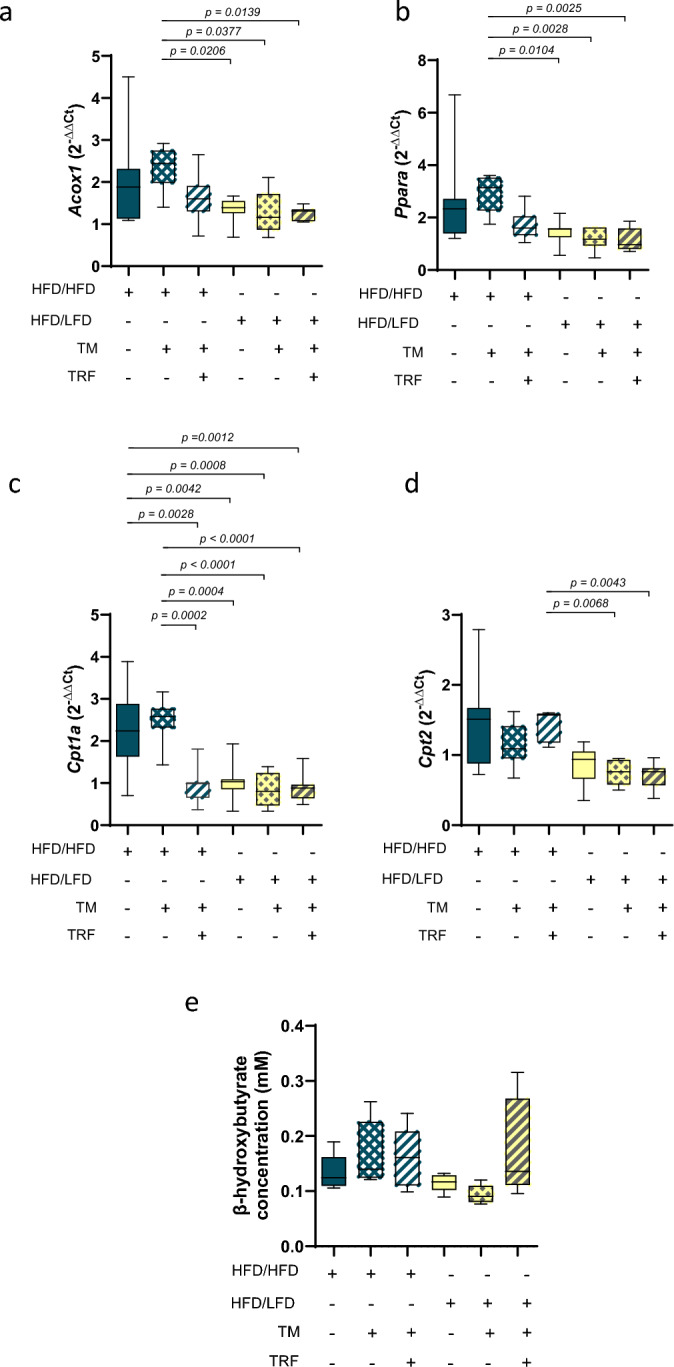
mRNA expression of β-oxidation-related genes. *Acox1* (**a**: HFD/HFD n = 7, HFD/HFD + TM n = 7, HFD/HFD + TM + TRF n = 7, HFD/LFD n = 7, HFD/LFD + TM n = 7, HFD/HFD + TM + TRF n = 7), *Ppara* (**b**: HFD/HFD n = 7, HFD/HFD + TM n = 7, HFD/HFD + TM + TRF n = 7, HFD/LFD n = 7, HFD/LFD + TM n = 7, HFD/HFD + TM + TRF n = 7), *Cpt1a* (**c**: HFD/HFD n = 7, HFD/HFD + TM n = 7, HFD/HFD + TM + TRF n = 7, HFD/LFD n = 7, HFD/LFD + TM n = 7, HFD/HFD + TM + TRF n = 7), *Cpt2* (**d**: HFD/HFD n = 7, HFD/HFD + TM n = 7, HFD/HFD + TM + TRF n = 7, HFD/LFD n = 7, HFD/LFD + TM n = 7, HFD/HFD + TM + TRF n = 7) and plasma concentration of β-hydroxybutyrate (**e**: HFD/HFD n = 5, HFD/HFD + TM n = 5, HFD/HFD + TM + TRF n = 5, HFD/LFD n = 5, HFD/LFD + TM n = 5, HFD/HFD + TM + TRF n = 5). Gene expressions were normalized against the housekeeping gene 40S ribosomal protein S18 (*Rps18*) as well as positive control (PC, C57BL6 liver pool) and relative quantification was carried out via the 2^-ΔΔCT^ method (first Δ against *Rps18* Ct values and second Δ against PC Ct values). Statistical significance was assessed using either one-way ANOVA followed by Tukey’s multiple comparisons test (**c**), Kruskal–Wallis test followed by Dunn’s post hoc test (**d**), or Brown-Forsythe and Welch ANOVA followed by Tamhane’s multiple comparisons test (**a**: F value (F) = 5.142, degrees of freedom (DF) = 5; **b**: F = 4.049, DF = 5; **e**: F = 2.010, DF = 5). Blue dots and box plots indicate HFD groups, yellow dots and box plots indicate diet change to LFD. The table below the figure displays the individual groups, respectively. Table is read from top to bottom, ‘ + ’ denotes implementation of a given diet or intervention and ‘−’ its absence. Data are presented as mean ± SD and statistical significance was set at p < 0.05. HFD: high-fat diet, LFD: low-fat diet, TM: treadmill, TRF: time-restricted feeding.

**Fig. 4:**
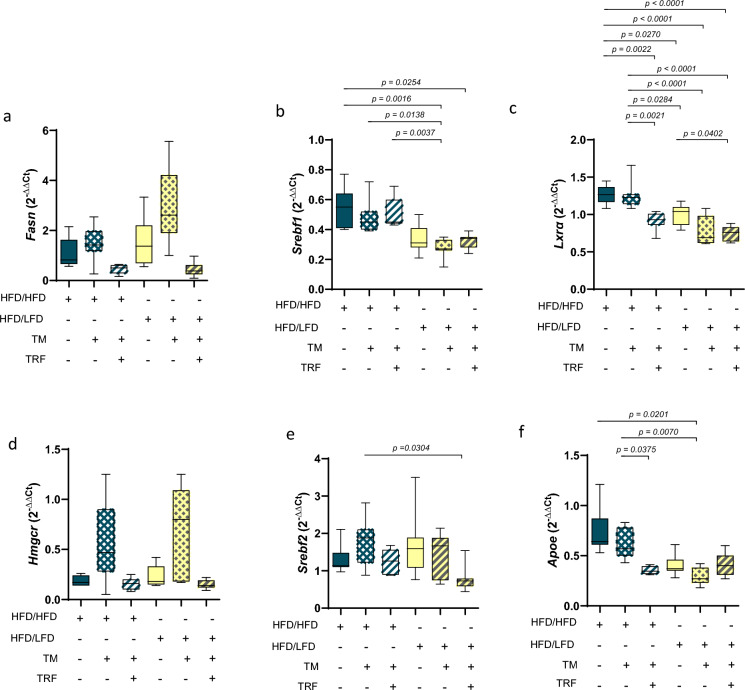
mRNA expression of lipogenesis- and cholesterol synthesis-related genes. *Fasn* (**a**: HFD/HFD n = 7, HFD/HFD + TM n = 7, HFD/HFD + TM + TRF n = 7, HFD/LFD n = 7, HFD/LFD + TM n = 7, HFD/LFD + TM + TRF n = 7), *Sreb1f.* (**b**: HFD/HFD n = 7, HFD/HFD + TM n = 7, HFD/HFD + TM + TRF n = 7, HFD/LFD n = 7, HFD/LFD + TM n = 7, HFD/LFD + TM + TRF n = 7), *Lxrα* (**c**: HFD/HFD n = 6, HFD/HFD + TM n = 7, HFD/HFD + TM + TRF n = 6, HFD/LFD n = 7, HFD/LFD + TM n = 7, HFD/LFD + TM + TRF n = 7), *Hmgcr* (**d**: HFD/HFD n = 7, HFD/HFD + TM n = 7, HFD/HFD + TM + TRF n = 7, HFD/LFD n = 7, HFD/LFD + TM n = 7, HFD/LFD + TM + TRF n = 7), *Srebf2* (**e**: HFD/HFD n = 7, HFD/HFD + TM n = 7, HFD/HFD + TM + TRF n = 7, HFD/LFD n = 7, HFD/LFD + TM n = 7, HFD/LFD + TM + TRF n = 7) and *Apoe* (**f**: HFD/HFD n = 7, HFD/HFD + TM n = 7, HFD/HFD + TM + TRF n = 6, HFD/LFD n = 7, HFD/LFD + TM n = 7, HFD/LFD + TM + TRF n = 7). Gene expressions were normalized against the housekeeping gene 40S ribosomal protein S18 (*Rps18*) as well as positive control (PC, C57BL6 liver pool) and relative quantification was carried out via the 2^-ΔΔCT^ method (first Δ against *Rps18* Ct values and second Δ against PC Ct values). Significance of differences between groups was tested either with Brown-Forsythe and Welch-ANOVA followed by Tamhanes´s multiple comparisons test, (**a**: F value (F) = 8.044, Degree of Freedom (DF) = 5, **d**: F = 6.495, DF = 5; **f**: DF = 5, F = 8.142, DF = 5), Kruskal–Wallis followed by Dunn´s multiple comparisons test (**b**) or One-way ANOVA followed by Tukey´s multiple comparisons test (**c**, **e**). Blue dots and box plots indicate HFD groups, yellow dots and box plots indicate diet change to LFD. The table below the figure displays the individual groups, respectively. Table is read from top to bottom, ‘ + ’ denotes implementation of a given diet or intervention and ‘−’ its absence. Data are presented as mean ± SD and statistical significance was set at p < 0.05. HFD: high-fat diet, LFD: low-fat diet, TM: treadmill, TRF: time-restricted feeding.

**Fig. 5:**
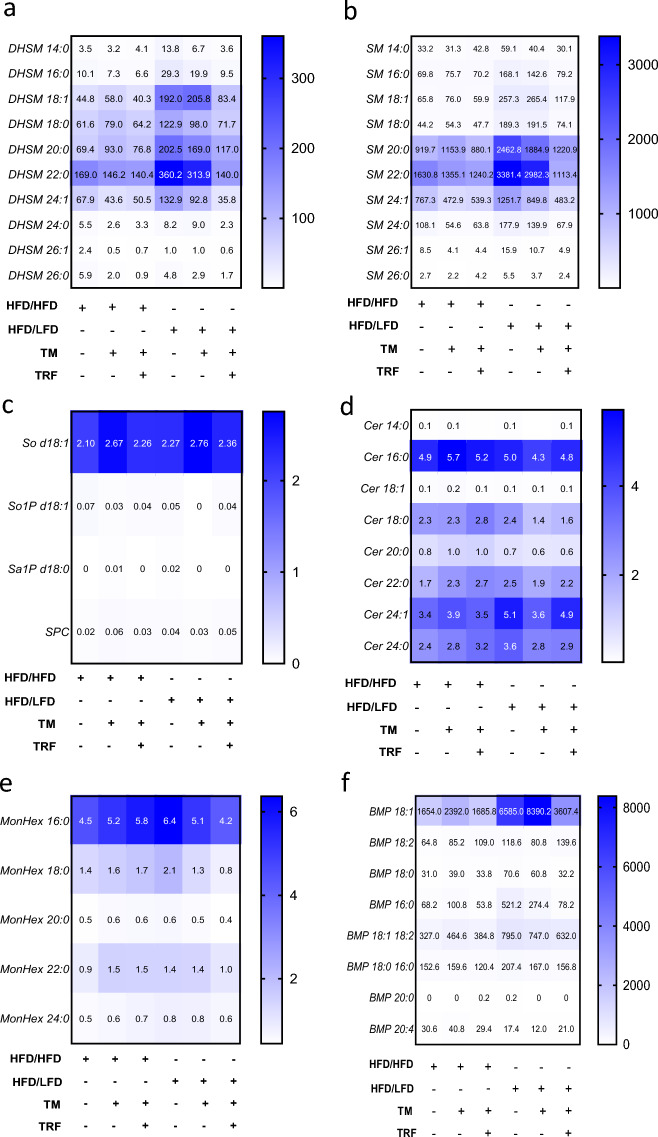
Heat maps depicting the relative abundance of dihydrosphingomyelins (**a**, DHSM), sphingomyelins (**b**, SM), glycosphingolipids (**c**), ceramides (**d**, Cer), monohexosylceramides (**e**, MonHex) and bis(monoacylglycerol)phosphate (**f**, BMP) in liver tissue (HFD/HFD n = 5, HFD/HFD + TM n = 5, HFD/HFD + TM + TRF n = 5, HFD/LFD n = 5, HFD/LFD + TM n = 5, HFD/HFD + TM + TRF n = 5). Lipid concentrations are quantified as pmol per mg of liver tissue. Color intensity reflects lipid abundance, with dark blue indicating high, light blue intermediate, and white low concentrations of the respective lipid species. Statistical significance was assessed using two-way ANOVA (lipid effect: **a:** p < 0.0001, F value (F) = 161.3, Degree of Freedom (DF) = 9; **b**: p < 0.0001, F = 223.3, DF = 9; **c**: p < 0.0001, F = 314.9, DF = 3; **d:** p < 0.0001, F = 270.7, DF = 7; **e**: p < 0.0001, F = 564.9, DF = 4; **f**: p < 0.0001, F = 173.5, DF = 7; intervention effect: **a**: p < 0.0001, F = 47.64, DF = 5; **b**: p < 0.0001, F = 34.85, DF = 5; **c**: p < 0.0001, F = 6.165, DF = 5; **d:** F = 0.5430, DF = 5; **e**: p < 0.0001, F = 12.51, DF = 5; **f**: p < 0.0001, F = 18.78, DF = 5 and interaction effect: **a**: p < 0.0001, F = 7.079, DF = 45; **b**: p < 0.0001, F = 8.738, DF = 45; **c**: p = 0.0030, F = 1.921, DF = 35; **d**: F = 0.6392, DF = 15; **e**: p < 0.0001, F = 2.637, DF = 20; **f**: p < 0.0001, F = 14.39, DF = 35; followed by Sidak or Tukey’s multiple comparisons test (p values in main text). Data are presented as mean and statistical significance was set at p < 0.05. HFD: high-fat diet, LFD: low-fat diet, TM: treadmill, TRF: time-restricted feeding. The table below the figure displays the individual groups, respectively. Table is read from top to bottom, ‘ + ’ denotes implementation of a given diet or intervention and ‘−’ its absence.

**Fig. 6:**
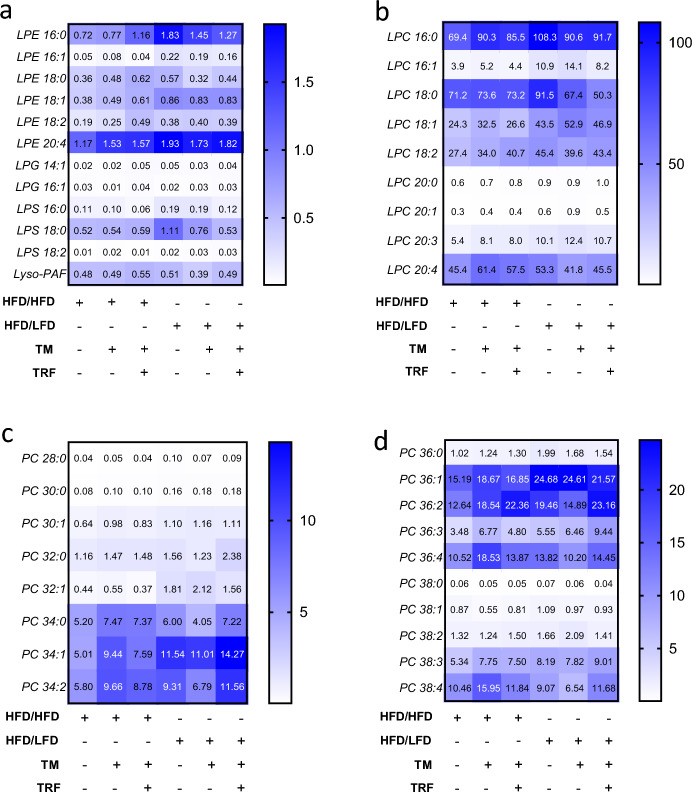
Heat maps depicting the relative abundance of lysophosphatidylethanolamines (**a**, LPE), lysophosphatidylcholines (**b**, LPC) and phosphocholines (**c** and **d,** PC). Lipid concentrations are quantified as pmol per mg of liver tissue (HFD/HFD n = 5, HFD/HFD + TM n = 5, HFD/HFD + TM + TRF n = 5, HFD/LFD n = 5, HFD/LFD + TM n = 5, HFD/HFD + TM + TRF n = 5). Color intensity reflects lipid abundance, with dark blue indicating high, light blue intermediate, and white low concentrations of the respective lipid species. Statistical significance was assessed using two-way ANOVA (lipid effect: **a**: p = 0.0001, F value (F) = 137.6, Degree of Freedom (DF) = 11, **b**: p < 0.0001, F = 428.4, DF = 8; **c**: p < 0.0001, F = 206.1, DF = 7; **d**: p < 0.0001, F = 203.7, DF = 9; intervention effect: **a**: p < 0.0001, F = 12.18, DF = 5, **b**: p < 0.0001, F = 10.97, DF = 5; **c**: p < 0.0001, F = 11.66, DF = 5; **d**: p < 0.0001, F = 8.216, DF = 5 and interaction effect: **a**: p < 0.0001, F = 2.002, DF = 55, **b**: p < 0.0001, F = 3.739, DF = 40); **c**: p < 0.0001, F = 3.696, DF = 35; **d**: p < 0.0001, F = 3.008, DF = 45) followed by Tukey’s multiple comparisons test. Data are presented as mean and statistical significance was set at p < 0.05. HFD: high-fat diet, LFD: low-fat diet, TM: treadmill, TRF: time-restricted feeding. The table below the figure displays the individual groups, respectively. Table is read from top to bottom, ‘ + ’ denotes implementation of a given diet or intervention and ‘−’ its absence.

Statistical analyses were performed using GraphPad Prism version 10.4.2. (GraphPad Software Inc., San Diego, CA, USA), as previously described by our group^28^. Results (Figs. 1, 2, 3 and 4) are presented as box plots displaying the median, 25th and 75th percentiles, and a 95% confidence interval. Outliers were identified and removed using the ROUT method based on a false discovery rate (Q = 0.01). For expression analyses, n = 7 samples were analysed; for the β-hydroxybutyrate assay, n = 5 samples were used. Normality was assessed using the Kolmogorov–Smirnov test for score data or the Shapiro–Wilk test. For normally distributed data, homogeneity of variances was tested using Bartlett’s test (p > 0.05). If variances were unequal or sample size too small, statistical significance was determined using Brown-Forsythe and Welch’s ANOVA, followed by Tamhane’s post hoc test. If variances were homogeneous, one-way ANOVA followed by Tukey’s multiple comparisons test was applied. For non-normally distributed data, the Kruskal–Wallis test followed by Dunn’s multiple comparisons test was used. All statistical tests were performed as two-tailed unless stated otherwise. Data are presented as mean ± standard deviation (SD). For further details, see figure legends or figures, respectively. For the heat map data (Figs. 5 and 6, each n = 5), a two-factor analysis was performed (factor 1: lipids; factor 2: intervention, p and F values, as well as degree of freedom (DF) are mentioned in the figure legends) using a two-way ANOVA, followed by Sidak’s or Tukey’s multiple-comparisons tests (p values are mentioned in the section “Results”). The data presented as mean values ± SD and the corresponding p-values are reported in the main text. The alpha level was set at 0.05, and statistical significance was considered at p < 0.05.

## Results

Prolonged HFD consumption resulted in a marked increase in body weight over the initial six months (Fig. 1d). Upon initiation of the intervention phase -including LFD, TM exercise, and TRF- only the dietary switch to LFD led to a marked reduction in body weight within a few weeks (Fig. 1d, yellow vs. blue). At the end of the study, the final body weight in the LFD groups was approximately -and thus significantly- 50% lower compared to all groups that remained on HFD (Fig. 1e, yellow vs. blue). In addition, the HFD combined with TM + TRF also tended to show a reduction in body weight compared to HFD alone, although this difference was not statistically significant (Fig. 1e).

Plasma AST, ALT, and albumin levels were measured to assess liver injury and function (Fig. 2). The results showed that the HFD group without intervention, or combined with TM alone, exhibited the highest AST and ALT levels reaching nearly 200 U/L and 150 U/L, respectively (Fig. 2a and b). In contrast, the combination with TRF, and especially the subsequent dietary change, significantly reduced these values to physiological ranges (Fig. 2a and b). Surprisingly, albumin levels with almost 38 ± 6 g/L were highest in the HFD group without intervention (Fig. 2c). In the HFD groups with interventions, albumin levels tended to be lower; however, this trend did not reach statistical significance. Considering the dietary switch, a significant increase is observed in the LFD group with TM and TRF compared to the HFD groups with TRF and/or TM (Fig. 2c).

After lipolysis, fatty acids undergo β-oxidation in peroxisomes and mitochondria^30^. Accordingly, we analyzed the gene expression of *Acox1* and *Ppara*, which are associated with β-oxidation in peroxisomes and mitochondria, as well as *Cpt1a* and *Cpt2*, which regulate important steps of fatty acid catabolism in mitochondria^14^ (Fig. 3a–d). All LFD groups showed significantly lower expression levels compared with the HFD + TM group (Figs. 3a and b). Interestingly, *Cpt1a* expression in the HFD + TM + TRF group resembled that of the LFD groups (Fig. 3c), a pattern not observed for *Cpt2* (Fig. 3d). Plasma β-hydroxybutyrate concentrations (Fig. 3e) remained largely unchanged after the intervention. Nevertheless, there was a trend -albeit not statistically significant- toward higher values in the LFD + TM + TRF group compared with both other LFD groups. This may suggest a subtle effect of intermittent fasting on ketone metabolism rather than reflecting increased ketogenesis.

Lipogenesis depends on fatty acid synthesis, with *Fasn* expression regulated by the transcription factors SREBP1 and LXRα^10^. In this context, a change in diet combined with TM exercise tended to increase *Fasn* expression (Fig. 4a), while the addition of TRF tended to decrease it, even in case of HFD continuation. *Sreb1f.* expression (Fig. 4b) was significantly reduced in the LFD group in combination with TRF and/or TM compared to HFD alone or HFD in combination with TRF and/or TM. Notably, *Lxrα* expression in the HFD + TM + TRF group was similar to that in all LFD groups and was significantly lower than in the HFD group alone (Fig. 4c).

Cholesterol synthesis, alongside fatty acid synthesis, is vital in lipid metabolism^10^. In light of this, *Hmgcr* expression (Fig. 4d) tended to be highest in the TM groups, regardless of diet. In terms of *Srebf2* expression, only the diet-change combined with TM and TRF showed a significant reduction compared to continued HFD combined with TM (Fig. 4e), suggesting that dietary modification together with intermittent fasting may exert the strongest suppressive effect on cholesterol-regulatory pathways. *Apoe* expression (Fig. 4f) was reduced in all LFD groups, reaching significance only in the LFD + TM group compared to HFD and HFD + TM, and was likewise decreased in the HFD + TM + TRF vs. HFD + TM group.

Based on these results, a hepatic lipid profile was generated by determination of sphingolipids (Fig. 5) as well as lysophospholipids and phospholipids (Fig. 6). Throughout all lipid analyses a strong lipid effect within each lipid class as well as an intervention effect (expect for Fig. 5c) was observed (for details please see figure legends). Notably, DHSM 22:0 (p < 0.0001, Fig. 5a), SM 22:0 (p < 0.001, Fig. 5b), and sphingosine d18:1 (p < 0.0001, Fig. 5c) were significantly elevated under HFD compared to all other DHSM, SM and sphingosine species. Following dietary intervention, alone or combined with TM exercise, DHSM 18:1, 20:0, and 22:0 (all p < 0.0001) as well as SM 20:0 and 22:0 (both p < 0.0001) increased significantly compared to HFD alone.

Cer and monohexosylceramide (MonHex) levels were significantly elevated under HFD only for the 16:0 species compared to all other Cer and MonHex species (Cer 16:0: p < 0.01, Fig. 5d; MonHex 16:0: p < 0.001, Fig. 5e). Cer 24:1 showed a modest increase following dietary change and TM combined with TRF, reaching significance only after the diet (p < 0.001 vs. HFD). Similarly, MonHex 16:0 responded significantly to the dietary intervention compared to HFD alone (p < 0.0001).

Regarding BMP analysis, comparison of BMP species within the HFD group revealed that only the 18:1 fatty acid was significantly elevated under HFD conditions (p < 0.01) compared with all other BMP species. After the dietary change and TM exercise, 18:1 levels increased significantly -by four- to five-fold- relative to HFD alone (p < 0.0001, Fig. 5f).

Examination of LPE species indicated that LPE 20:4 exhibited the most pronounced rise relative to all other LPE species under HFD (Fig. 6a, p < 0.01, except vs. 16:0). All three interventions led to a marked increase of LPE 16:0 (p < 0.01) and LPE 20:4 (p < 0.01) compared to HFD alone, with TM and TRF specifically enhancing LPE 16:0 species under continued HFD (p < 0.05).

Assessment of LPC species (Fig. 6b) showed significant elevations of LPC 16:0, 18:0, and 20:4 in all HFD groups relative to all LPCs (p < 0.05). Under continued HFD, TM and TRF specifically raised LPC 16:0 levels (p < 0.05 vs. HFD/HFD, Fig. 6b). Following the dietary modification, only LPC 16:0 (p < 0.0001) and 18:0 (p < 0.01) exhibited further significant increases vs. HFD alone.

PC analysis (Fig. 6c and d) revealed significant increase of PC 34:0, 34:1, 34:2 (Fig. 6c, p < 0.01) and PC 36:1, 36:2, 36:4, 38:4 (Fig. 6d, p < 0.01) under HFD compared to all other PC species. Under HFD combined with TRF or TM, PC 34:1, 34:2, 36:2, 36:4, and 38:4 species were significantly increased (p < 0.05). Upon LFD combined with TRF and/or TM, only PC 34:1 and 36:1 showed a significant rise within the PC class (p < 0.001, Fig. 6c and d).

Correlation analyses (Figure S1a-f and Figure S2a-f) demonstrated strong positive associations among the selected lipids, which were chosen based on their significant regulation (see Fig. 5 and 6). For example, DHSM 20:0 and SM 20:0 correlated almost perfectly (r = 0.99). Similarly, lipogenic genes and genes involved in β-oxidation showed strong positive correlations among themselves, for example *Sreb1f* and *Lxra* correlating with r = 0.79 and *Ppara* and *Cpt1a* with r = 0.97. Correlation analysis further showed pronounced negative correlations between lipid species and markers of liver function (AST and ALT). For example, DHSM 20:0 correlated negatively with AST (r =  − 0.70), core lipogenic genes such as *Lxra* (r =  − 0.74) and *Apoe* (r =  − 0.81), and the β-oxidation gene *Cpt1a* (r =  − 0.72). Comparable relationships were observed for ceramides, particularly Cer 24:1, which negatively correlated with AST (r =  − 0.70), *Lxra* (r =  − 0.83), and *Cpt1a* (r =  − 0.69). Among lysophospholipids, for example LPC 16:0 showed a strong negative correlation with AST (r =  − 0.77). Furthermore, LPE 20:4 and LPC 16:0 were strongly negatively correlated with lipogenic genes (*Lxra* and *Apoe*) as well as with all β-oxidation-related genes studied. Strong negative correlations with liver function markers, lipogenic genes, and β-oxidation-related genes were also observed for PC 34:1 and PC 36:1. Additionally, lipogenic and β-oxidation-related genes were positively correlated with each other (e.g., *Lxra* and *Cpt1a*, r = 0.9; Figure S3a-d) when all these genes were correlated with all relevant lipid species suggesting a comparable contribution of both pathways.

## Discussion

Metabolic syndrome refers to the concurrent presence of multiple clinical conditions and risk factors that together significantly increase the likelihood of developing serious metabolic and cardiovascular diseases. The syndrome is primarily driven by unhealthy lifestyle factors, particularly physical inactivity and excessive or imbalanced dietary intake. In this study, we investigated hepatic gene expression of lipid metabolism and liver function under varying dietary conditions and exercise regimens.

As expected, prolonged consumption of a HFD resulted in significant weight gain, confirming its obesogenic potential^31–33^. Switching to a LFD led to rapid and sustained weight loss, ultimately reducing final body weight by approximately 50% compared to animals maintained on HFD. This aligns with previous intervention studies demonstrating effective weight normalization following dietary fat reduction^25,34^.

Neither TM exercise alone nor TRF under continuous HFD conditions led to significant weight loss, underscoring the dominant role of dietary composition in long-term body weight regulation^35^. However, the combination of exercise and TRF during the active (dark) phase resulted in ~ 20% weight reduction despite continued HFD consumption, emphasizing the metabolic benefits of circadian-aligned feeding. These findings support evidence that TRF can improve metabolic outcomes independently of caloric intake^36^, likely through enhanced mitochondrial rhythmicity and fatty acid oxidation, which increase energy expenditure and reduce fat accumulation^37^.

This pattern was mirrored in markers of liver injury. HFD feeding led to elevated levels of the liver enzymes AST and ALT, indicative of hepatocellular damage. Both TM and TRF significantly reduced these enzyme levels. This observation is unlikely to be solely attributable to reduced food intake, as food intake itself may affect liver enzyme levels^38^. Furthermore, because TRF was applied during the active phase -when mice naturally consume the majority of their daily food- the benefits appear to arise from the timing of feeding rather than from caloric restriction as also described by Hatori et al.^36^. Comparable improvements were observed following the dietary switch to LFD, although additional exercise or fasting interventions did not further enhance these effects. These findings reflect reduced hepatic injury and are consistent with prior studies demonstrating the reversibility of early-stage MASLD through dietary intervention^27,39,40^. Moreover, these biochemical improvements correlated with reductions in histological MASH scores as shown by our group in previous study^27^, here described as NASH score].

Interestingly, albumin levels appeared slightly higher in the HFD-only group and showed a decrease upon intervention with TM and TRF; although this observation was not statistically significant, it can be interpreted in the context of normal physiological variability. Since albumin usually only decreases in advanced liver disease, the slight decline observed in TM and TRF with continued HFD likely reflects normal fluctuations in liver protein metabolism rather than significant changes in liver function, as serum albumin usually remains stable until the later stages of liver dysfunction^41,42^. Following the dietary change combined with TM and TRF, albumin levels increased significantly, which may indicate an improvement in hepatic functional capacity, as serum albumin is a well-established marker of hepatic synthetic function and rises to normal values may be interpreted as enhanced liver performance^40^.

Consistent with prior findings showing elevated levels of leptin, LDL, HDL, total cholesterol, and TGs in HFD-fed mice^27^, our lipidomic analyses revealed pronounced alterations in hepatic lipid profiles under HFD, confirming earlier results^43^. Notably, in particular DHSM 22:0 and SM 22:0 species were elevated under HFD and further increased after dietary switch combined with exercise, suggesting membrane lipid remodeling during hepatic recovery. This aligns with the proposed role of SM in lipid raft formation and membrane organization^44^. In this context, the observed increases in SM and DHSM -particularly under LFD and in combination with TM- are more likely to reflect adaptive physiological responses rather than pathological lipid accumulation, as the reduced MASH score suggests [Ref.^27^, here still described as NASH score]. Furthermore, these lipid changes appear to support energy homeostasis and lipid transport, suggesting a regulated metabolic adaptation rather than a dysregulation of lipid metabolism^45–47^. This is consistent with a reduction in liver injury, as indicated by correlation analyses showing a negative relationship between lipid content and liver injury markers. In line with this interpretation, switching to a LFD reduced hepatic TG levels, whereas TM alone transiently increased TG levels regardless of diet as reported by Power Guerra et al.^27^. This effect may reflect enhanced hepatic TGs synthesis and VLDL secretion following exercise^48,49^, ultimately contributing to lipid mobilization and fat removal -consistent with the observed reduction in hepatic fat accumulation^27^.

In addition, Cer (particularly Cer 16:0) levels were elevated in HFD, which is consistent with previous findings^17^. Upon interventions, Cer 16:0 levels remained largely unchanged, while Cer 24:1 increased only after the dietary intervention. These findings suggest that Cer are unlikely to be the primary mediators of the lipid-lowering effects of the interventions. Instead, SM species, which were also elevated in this study, serve as the main reservoir for inducible Cer synthesis^17^ and thus contribute to the dynamic regulation of hepatic lipid metabolism. Regarding BMP fatty acids, only BMP 18:1 was elevated under HFD and could be further increased by intervention, potentially reflecting enhanced lysosomal function and lipid clearance^18^. In summary, these results indicate that lifestyle changes can partially reverse the disturbances in liver lipid metabolism characteristic of early-stage MASLD^21–23^.

In addition, some LPC and LPE species (e.g., LPC 16:0, 20:4) were markedly elevated under HFD and further increased, particularly after dietary switch. These changes likely represent compensatory phospholipid remodeling in response to lipid turnover or cellular repair. Elevated LPC levels are commonly linked to ER stress and hepatotoxicity, whereas increases in LPE and PC are associated with beneficial membrane remodeling^43^. Supporting this, PC species -particularly PC 34:1, 34:2, 36:2, 36:4, and 38:4- were elevated under HFD combined with TRF or TM, and continued to rise after dietary intervention. Given their protective role in HFD-induced obesity and associated complications such as hyperlipidemia and MASLD^50^, the observed increases in PC species likely reflect a beneficial metabolic adaptation with improved liver function, which is further supported by the negative correlation between AST and ALT levels and PC species.

At the molecular level, gene expression analysis revealed that β-oxidation-related genes (e.g., *Acox1, Ppara, Cpt1a*) were particularly downregulated upon intervention on LFD when compared to HFD + TM group. In the continuous HFD group, combined TRF and TM training reduced *Cpt1a* expression to levels comparable with those in LFD groups, whereas *Cpt2* remained unchanged, consistent with the lack of detectable changes in β-oxidation. This pattern may reflects the CPT system, in which CPT1A mediates the rate-limiting entry of long-chain fatty acids into mitochondria, while CPT2 regenerates CoA from acylcarnitines for β-oxidation^51–53^. *Cpt2*, located downstream within the mitochondrial matrix, may be more strongly influenced by sustained lipid flux and mitochondrial fatty acid load under HFD conditions, rendering it less responsive to this combined intervention. Moreover, these findings indicate that not only dietary changes, but particularly exercise and intermittent fasting, reduce the need for compensatory oxidative pathways, primarily in specific parts of the β-oxidation network, likely reflecting a partial stabilization of hepatic metabolic activity. These findings indicate that fat reduction through these interventions exerts distinct modulatory effects on hepatic lipid metabolism, consistent with previous pharmacological studies demonstrating selective regulation of oxidative pathways^54^. Specifically, enhanced β-oxidation appears to contribute to the reduction of hepatic lipid content, while de novo fatty acid synthesis may also occur, as supported by the given data in the LFD groups. Overall, these results suggest that changes in hepatic lipid levels likely reflect a balance between fatty acid oxidation and synthesis, rather than being driven solely by β-oxidation, as no significant differences were observed in this pathway in the present study. Furthermore, it is also worth noting that in our previous work^27^, a reduction of TG levels was observed following a dietary switch combined with TM and TRF. This further suggests enhanced lipolysis, which is consistent with the observed decrease in hepatic steatosis, supporting the notion that in particular dietary change combined with TRF and TM promote normalization of hepatic lipid metabolism. *Acox1* and *Ppara* expression levels showed low values in all LFD groups, but significant higher values in the HFD + TM group, probably indicating increased lipid oxidation in response to sustained lipid overload^48^. This interpretation is further supported by our previous findings of elevated TG levels in this group^27^.

Expression of lipogenesis-associated genes (*Srebf1, Lxrα*) exhibited a modest decline under HFD combined with TM and TRF, reaching statistical significance only for *Lxrα*. Following dietary modification, a more consistent reduction was observed. These findings align with Vieira et al.^55^ and, to some extent, with Damasceno de Lima et al.^37^, although the latter primarily focused on food restriction rather than changes in diet composition. Overall, the results indicate reduced lipogenic activity, suggesting that all interventions contribute to reestablishing the metabolic balance between lipid synthesis and degradation^56^. However, genes involved in fatty acid and cholesterol biosynthesis (*Fasn*, *Hmgcr*) showed a non-significant trend toward upregulation in the LFD + TM group. Although this pattern could be consistent with altered energy demand or membrane turnover in response to the combined dietary and exercise intervention^37,49^, the lack of statistical significance precludes definitive conclusions. Therefore, these findings should be considered exploratory and warrant further investigation in adequately powered studies.

In addition, hepatic *Apoe* expression, a key regulator of VLDL secretion and lipid transport^57^, was significantly reduced after dietary change combined with exercise, and most prominently in the HFD + TM + TRF group, indicating that dietary modification is not strictly required to modulate this cholesterol transporter. Instead, the combination of physical activity and intermittent fasting appears sufficient to induce a comparable effect even under continued HFD conditions. Moreover, *ApoE* expression seems tightly linked to hepatic lipid content and may serve as a dynamic marker of MASLD severity, consistent with previous reports^27,57,58^.

Overall, the alterations in gene expression point toward a reprogramming of hepatic lipid metabolism rather than a straightforward suppression of β-oxidation or lipogenic pathways. Correlation analyses reveal inverse associations between hepatic lipid species and the expression of genes involved in both fatty acid oxidation and lipid synthesis. At the same time, these genes -irrespective of whether they are linked to β-oxidation or lipogenesis- are positively correlated with one another, indicating coordinated transcriptional regulation rather than reciprocal control. Together, this pattern suggests that lipid accumulation may reflect an adaptive or reparative remodeling process -similar to what has been described for sphingomyelin^44^—occurring in parallel with a concerted downregulation of genes governing hepatic lipid metabolic flux.

## Limitation

Overall, the observed changes in gene expression related to lipid metabolism should be interpreted with caution, as our data are correlational and do not provide mechanistic insights. Transcriptional alterations do not necessarily translate into functional protein changes, and the lipid shifts likely result from complex regulatory processes. Future studies employing mechanistic approaches are needed to clarify causal relationships between gene expression, lipid metabolism, and metabolic outcomes.

Another limitation of the present study is that only female mice were included. This choice was made to facilitate group housing and maintain consistent conditions for behavioral analyses, which were part of a broader intervention study previously published^28^. While this design ensured reproducible experimental conditions, it restricts the generalizability of our findings to male mice, and potential sex-specific differences in metabolic and hepatic responses remain to be addressed.

Finally, no time-series or dose–response analyses were conducted. The TRF intervention was applied between 9 and 12 months of age to target established obesity and early-stage hepatic steatosis, allowing assessment of reversal rather than prevention of metabolic alterations. Investigating the effects of different intervention timings, durations, or intensities could provide insights into potential joint or cumulative effects, which remain important questions for future research.

## Conclusion

Our findings demonstrate that dietary normalization is a major contributor to reversing obesity-induced alterations in liver function, lipid metabolism, and transcriptional regulation of lipid homeostasis. By integrating detailed lipidomics profiling with analyses of combined lifestyle interventions, we provide extensive descriptive, phenotypic data indicating that exercise and intermittent fasting may confer additional metabolic benefits even under sustained HFD exposure. These effects are reflected in distinct changes in hepatic lipid composition and gene expression associated with improvements in liver phenotype, including reduced hepatic steatosis. Nevertheless, it should be clearly emphasized that the study with its phenotypic data is exploratory and hypothesis-generating. The results highlight candidate molecular targets, such as *Cpt1a*, for future mechanistic studies. To elaborate causal relationships between lifestyle interventions and hepatic lipid metabolism, targeted knockout models (e.g., LXR or PPARα) and protein function analyses could be employed in upcoming studies, enabling direct testing of whether specific transcriptional regulators mediate the observed effects. Integrating lipidomics, transcriptomics, histopathology, and mechanistic knockout approaches in future studies will help clarify causal pathways and further delineate the molecular mechanisms underlying the reversal of early-stage MASLD.

## Supplementary Information

Supplementary Information 1.

Supplementary Information 2.

## Data Availability

All data supporting the findings of this study are available from the corresponding author upon reasonable request.
