# Supplementary material for: Exercise and time-restricted and/or dietary feeding jointly improve hepatic lipid homeostasis in diet-induced obese mice
Source: Sci Rep. 2026 Mar 25;16:10508. doi: 10.1038/s41598-026-45394-4 (PMC13031764; doi:10.1038/s41598-026-45394-4)
Supplement: Supplementary file 1 — Supplementary Information 1. [file 41598_2026_45394_MOESM1_ESM.pdf]

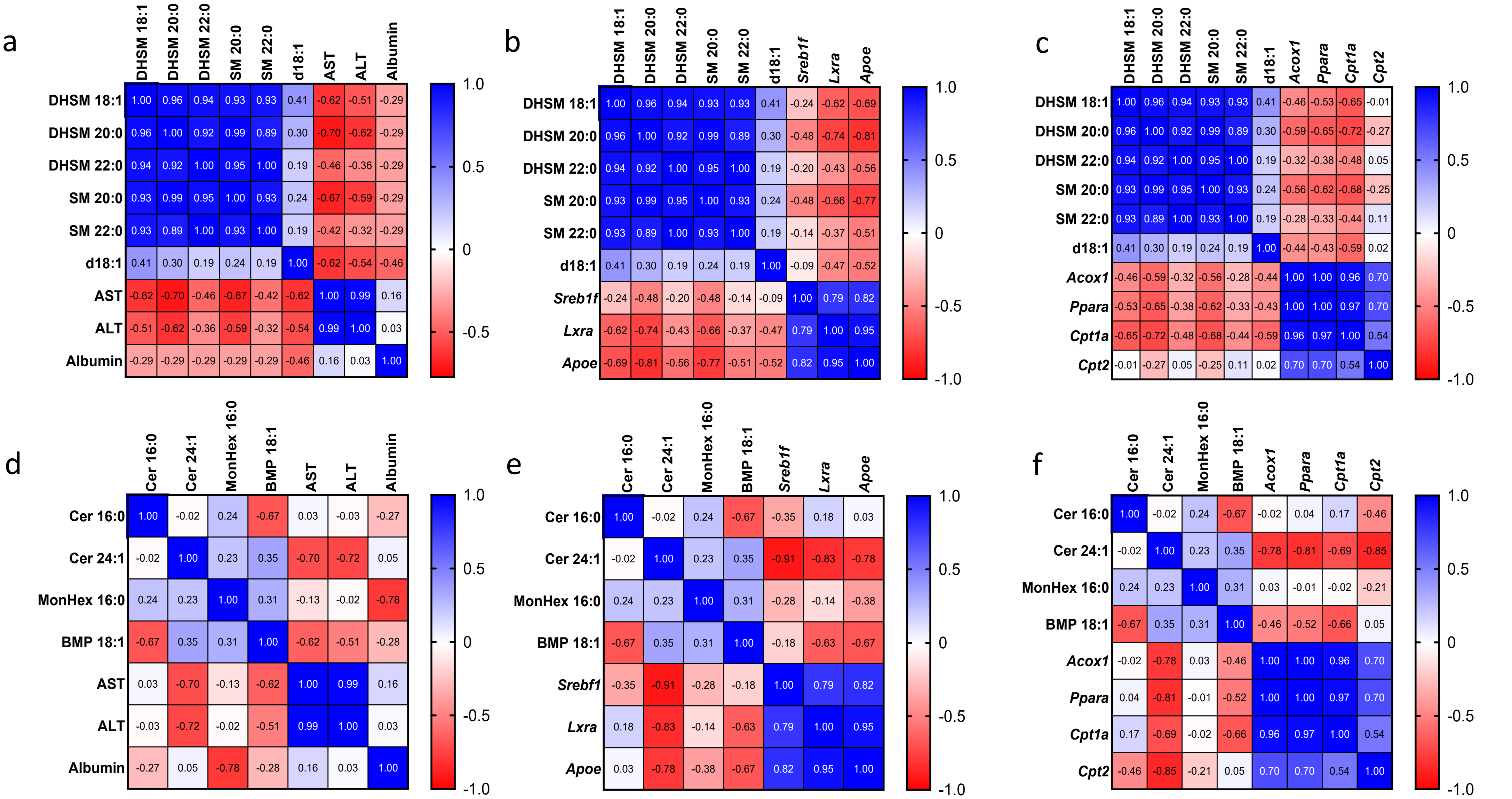

Lipids & liver function

Lipids & core lipogenic genes

Lipids & β-oxidation-related genes

Fig. S1

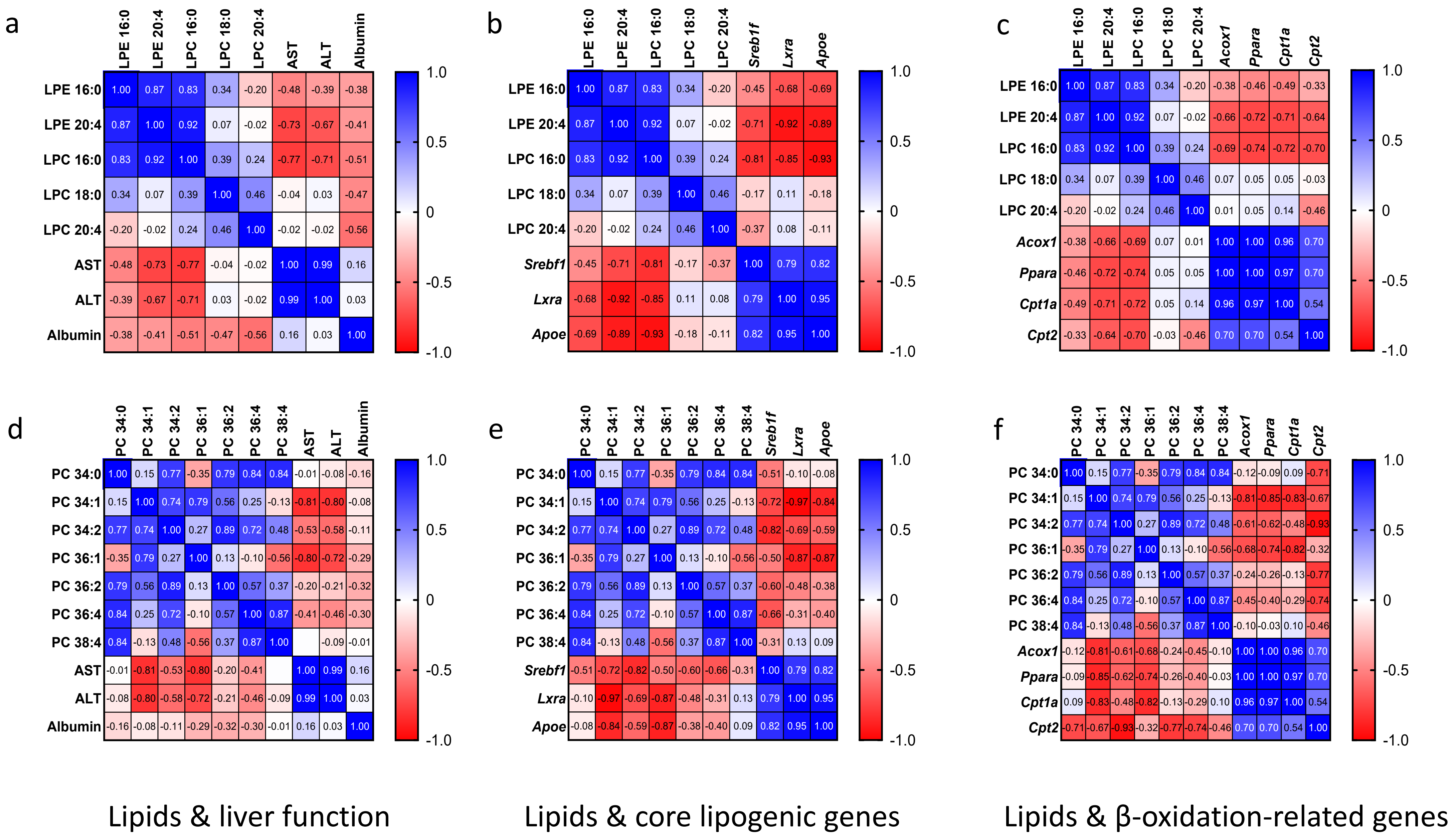
a
b
c
d
e
f
Lipids & liver function
Lipids & core lipogenic genes
Lipids & β-oxidation-related genes

Fig. S2

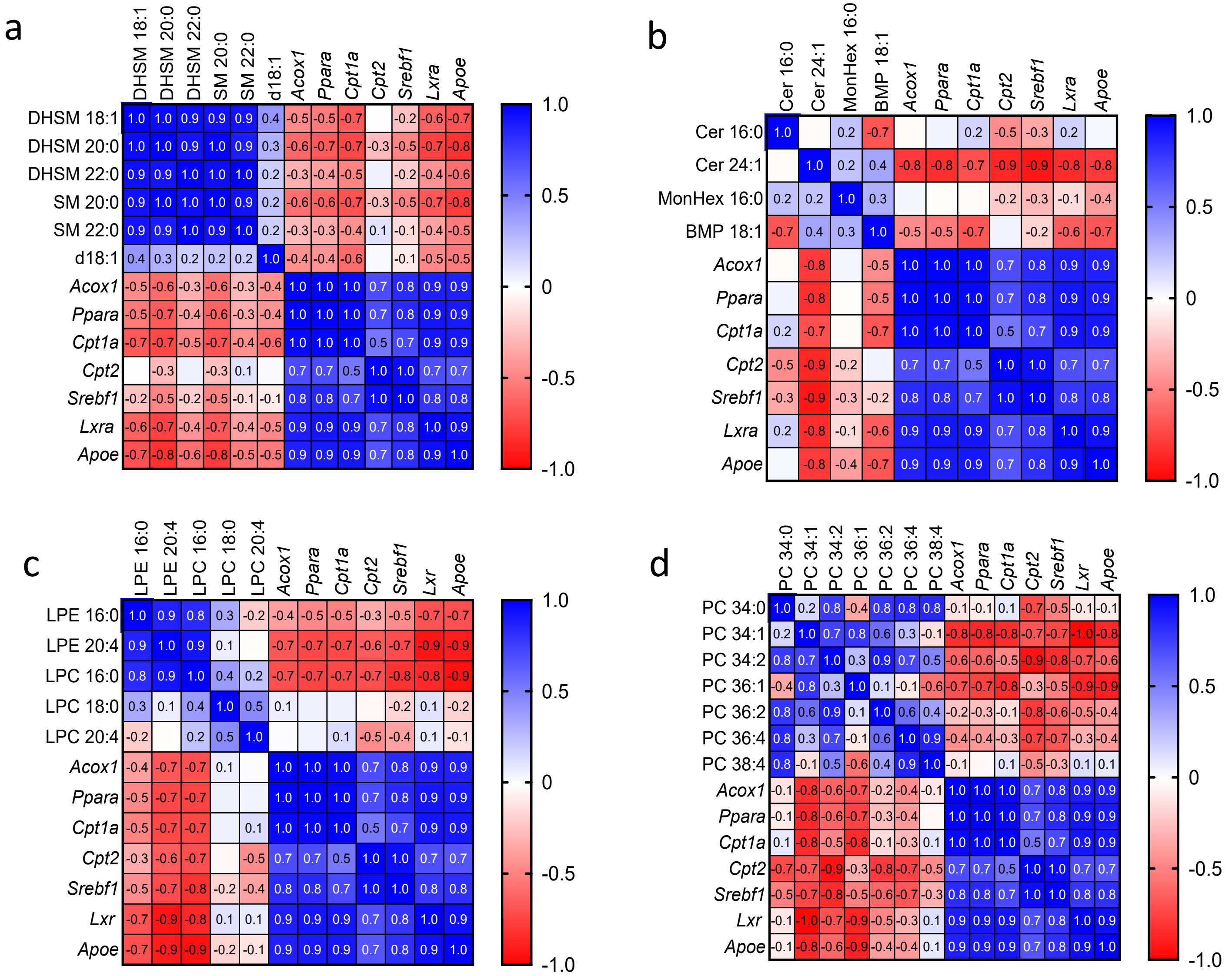

Fig. S3
