# Supplementary material for: Exercise and time-restricted and/or dietary feeding jointly improve hepatic lipid homeostasis in diet-induced obese mice
Source: Sci Rep. 2026 Mar 25;16:10508. doi: 10.1038/s41598-026-45394-4 (PMC13031764; doi:10.1038/s41598-026-45394-4)
Supplement: Supplementary file 2 — Supplementary Information 2. [file 41598_2026_45394_MOESM2_ESM.docx]

**Figures S1 (a-f):** Pearson correlation heat maps displaying correlation coefficients (r) between mean values of hepatic lipid species (dihydrosphingomyelins (DHSM), sphingomyelins (SM), glycosphingolipids, ceramides (Cer), monohexosylceramides (MonHex) and bis(monoacylglycerol)phosphate (BMP)), liver function markers (AST, ALT, albumin), core lipogenic genes (*Sreb1f*, *Lxra*, *Apoe*), and β-oxidation-related genes (*Acox1*, *Ppara*, *Cpt1a*, *Cpt2*). Strong positive correlations (r = 0.70–1.00) are shown in blue, strong negative correlations (r = −1.00 to −0.70) in red, and lighter colors indicate moderate correlations (r > 0.40 or r < −0.40).

**Figure S2 (a-f):** Pearson correlation heat maps displaying correlation coefficients (r) between mean values of hepatic lipid species (lysophosphatidylethanolamines (LPE), lysophosphatidylcholines (LPC) and phosphocholines (PC)), liver function markers (AST, ALT, albumin), core lipogenic genes (*Sreb1f, Lxra, Apoe*), and β-oxidation-related genes (*Acox1*, *Ppara, Cpt1a, Cpt2*). Strong positive correlations (r = 0.70–1.00) are shown in blue, strong negative correlations (r = −1.00 to −0.70) in red, and lighter colors indicate moderate correlations (r > 0.40 or r < −0.40).

**Figure S3 (a-d):** Pearson correlation heat maps displaying correlation coefficients (r) between mean values of hepatic lipid species (dihydrosphingomyelins (DHSM), sphingomyelins (SM), glycosphingolipids, ceramides (Cer), monohexosylceramides (MonHex) and bis(monoacylglycerol)phosphate (BMP), (lysophosphatidylethanolamines (LPE), lysophosphatidylcholines (LPC) and phosphocholines (PC)), core β-oxidation-related genes (*Acox1*, *Ppara, Cpt1a, Cpt2*) as well as lipogenic genes (*Sreb1f, Lxra, Apoe*). Strong positive correlations (r = 0.70–1.00) are shown in blue, strong negative correlations (r = −1.00 to −0.70) in red, and lighter colors indicate moderate correlations (r > 0.40 or r < −0.40).
